# Roles of perivascular adipose tissue in the pathogenesis of atherosclerosis ― an update on recent findings

**DOI:** 10.3389/fphys.2024.1522471

**Published:** 2025-01-06

**Authors:** Tomoya Hara, Masataka Sata

**Affiliations:** Department of Cardiovascular Medicine, Tokushima University Graduate School of Biomedical Sciences, Tokushima, Japan

**Keywords:** perivascular adipose tissue (PVAT), epicardial adipose tissue (EAT), vasa vasorum (VV), chronic inflammation, atherosclerosis, cardiovascular disease (CVD)

## Abstract

Lifestyle-related diseases, such as atherosclerosis and diabetes, are now considered to be a series of diseases caused by chronic inflammation. Adipose tissue is considered to be an endocrine organ that not only plays a role in lipid storage, heat production, and buffering, but also produces physiologically active substances and is involved in chronic inflammation. Perivascular adipose tissue (PVAT) surrounding blood vessels similarly produces inflammatory and anti-inflammatory physiologically active substances that act on blood vessels either directly or via the bloodstream. Epicardial adipose tissue (EAT), which is in direct contact with the coronary arteries inside the pericardium, is thought to have a direct effect on the coronary arteries as well. The presence and inflammatory status of these adipose tissues can be evaluated by imaging tests, and has been shown to be associated with the presence of current cardiovascular disease (CVD) and to be a prognostic factor. It is also expected to become a new diagnostic and therapeutic target for CVD.

## 1 Introduction

Cardiovascular disease (CVD), diabetes, and chronic kidney disease are recognized to be based on chronic inflammation (Furman et al., 2019). In addition, adipose tissue has recently been shown to not only play a role in energy storage, heat production, and cushioning between tissues, but also as an endocrine organ, secreting inflammatory and anti-inflammatory physiologically active substances that are involved in chronic inflammation (Matsuzawa et al., 2004). Recently, several reports have suggested a relationship between epicardial adipose tissue (EAT) and CVD (Tanaka et al., 2020; Wang et al., 2022). In 2023, the European Society of Cardiology reported a statement on the use of EAT as a therapeutic indicator and biomarker in clinical practice (Antoniades et al., 2023). This article mainly reviews the relationship between CVD and EAT and its potential as a therapeutic target.

## 2 Terminological definition of perivascular fat

**FIGURE 1:**
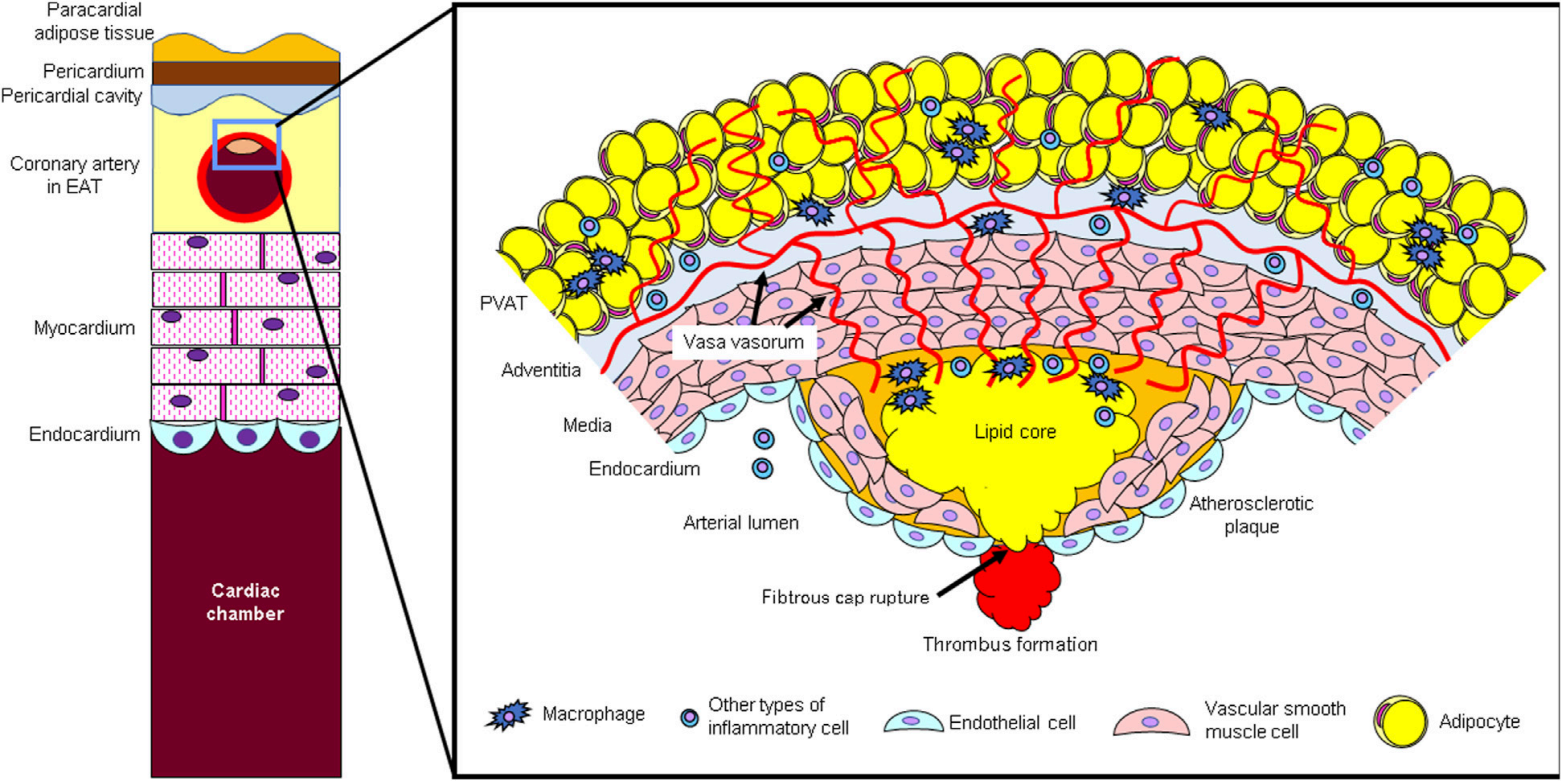
(Left side) Schematic diagram of epicardial adipose tissue (EAT) and coronary arteries. Epicardial adipose tissue (EAT) is located in the pericardial cavity, directly in contact with the coronary arteries, and adjacent to the myocardium. (Right side) Atherosclerotic lesion and perivascular adipose tissue (PVAT). PVAT secretes inflammatory cytokines. Bioactive agents secreted from PVAT act not only directly on blood vessels but also through adventitial vasa vasorum (VV).

A proposal by the European Society of Cardiology defined the names of each adipose tissue in 2023 (Antoniades et al., 2023) (Left side of Figure 1). Thoracic visceral adipose tissue (VAT) includes the EAT, which is enclosed between the cardiac surface and the visceral pericardium, the pericardial adipose tissue (external to the pericardium and surrounding the cardiac silhouette), as well as the non-pericardial thoracic adipose tissue (located anywhere inside the thoracic cavity, but outside the pericardium) (Antonopoulos and Antoniades, 2017). Perivascular adipose tissue (PVAT) around coronary arteries has distinct biological properties compared to the rest of the EAT far from the arterial wall (Antonopoulos et al., 2017; Costa et al., 2018; Antoniades et al., 2020; Turaihi et al., 2020). However, within EAT there is a gradual transition between PVAT and non-PVAT, with no anatomical structures separating the two, and a definition must be agreed upon. Indeed, in recent literature, PVAT has been defined as a layer of adipose tissue located within a distance equal to the luminal diameter of the artery, and this definition was adopted by the working group (Antoniades et al., 2020). This definition applies to PVAT surrounding human arteries with a lumen diameter of up to 2 cm. For arteries with a lumen diameter greater than 2 cm (such as the aorta), PVAT extends up to 2 cm from the external surface of the vessel.

## 3 Adventitial environment of blood vessels with atherosclerotic lesions

**FIGURE 2:**
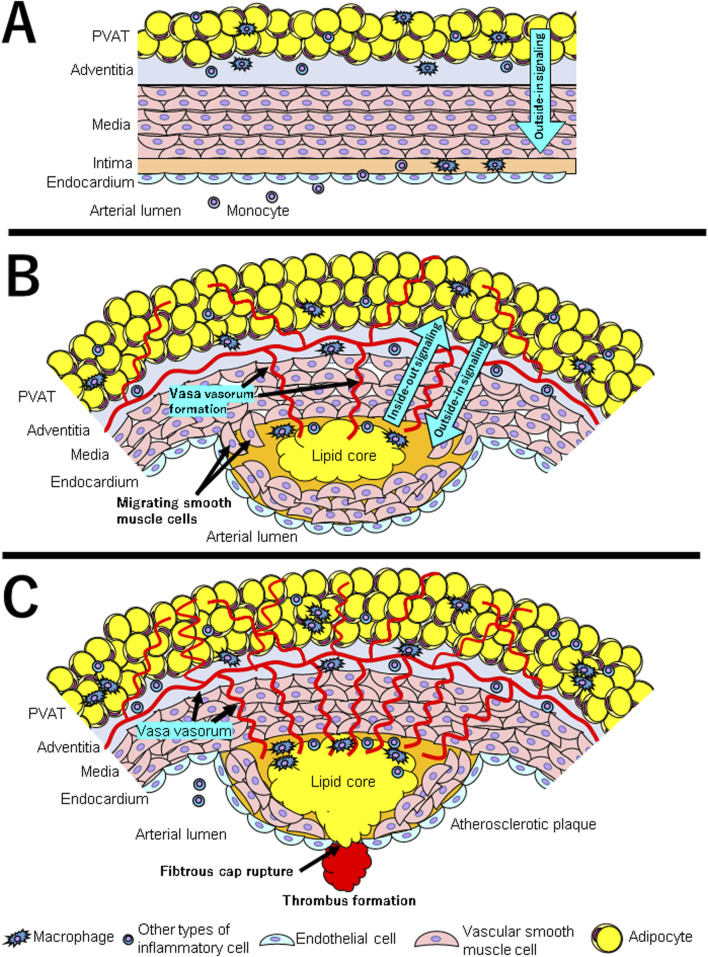
**(A–C)** show the interaction between PVAT and vascular wall at each stage in the development of atherosclerosis. **(A)** The first steps include adhesion of blood leukocytes to a monolayer of activated endothelial cells, migration of bound leukocytes to the intima, maturation of monocytes into macrophages and their lipid uptake to generate foam cells. Under normal circumstances, PVAT induces vasodilation and anti-inflammatory effects through the release of adipokines such as adiponectin and vasodilators such as nitric oxide (displayed as “Outside-in Signaling” with blue arrow). **(B)** Under inflammatory conditions such as obesity, PVAT produces adipokines such as leptin, which induces vasoconstriction, smooth muscle cell migration, and endothelial dysfunction. Pro-inflammatory cytokines are produced by PVAT macrophages and other inflammatory cells. At the same time, vasa vasorum is formed and invaded into intima. Inflammatory cytokines and chemokines produced by PVAT either infiltrate directly into the intima or enter through vasa vasorum (displayed as “Outside-in Signaling” with blue arrow). The inflammatory condition of vessel wall influences PVAT through paracrine signals that cause changes in the PVAT secretion phenotype (displayed as “Inside-out signaling” with blue arrow). **(C)** As a result of the interaction between chronic inflammatory condition in both PVAT and vascular wall, the atherosclerotic plaques gradually become unstable. Inflammation is a major factor in the thinning of the fibrous cap and the rupture of atherosclerotic plaques. Thrombosis complicates physical disruption of the atherosclerotic plaque. Fracture of the cap exposes blood coagulant components to tissue factors in the plaque, triggering occlusive thrombus formation that limits blood flow.

Atherosclerosis is caused when the homeostatic function of vascular endothelial cells is impaired and inflammatory cells infiltrate under the endothelium (Ross, 1999) (Figure 2A). Therefore, it has been thought that inflammation occurs from the luminal side and spreads to the adventitial side. However, more recent findings suggest a pathway through which inflammation on the adventitial side spreads to the lumen side (Figures 2B, C).

Most arteries, except cerebral arteries and microvessels, are surrounded by perivascular adipose tissue (PVAT) (Szasz et al., 2013). Although PVAT has been thought to act as a mechanical cushion for supporting tissues and the vasculature, recent studies have shown that PVAT secretes adipokines, including inflammatory cytokines and chemokines. Under normal circumstances, PVAT induces vasodilation and anti-inflammatory effects through the release of adipokines (such as adiponectin) and vasodilators (such as nitric oxide) (Akoumianakis et al., 2017) (Figure 2A). Under inflammatory conditions such as obesity, PVAT produces adipokines such as leptin, which induces vasoconstriction, SMC migration, and endothelial dysfunction (Kim et al., 2020; Ichikawa et al., 2023). Pro-inflammatory cytokines are produced by PVAT macrophages and other inflammatory cells. Therefore, they cause endothelial dysfunction and atherosclerotic plaque formation (Chen et al., 2010) (Figure 2B). Recent evidence also supports that the relationship between PVAT and vessel walls is bidirectional. This is because the inflammatory condition of vessel wall influences PVAT through paracrine signals that cause changes in the PVAT secretion phenotype (Oikonomou and Antoniades, 2019).

Accumulating evidence highlights the role of adipocytes as secreting cells of exosomes that convey miRNAs with either pro-atherosclerotic or anti-atherosclerotic effects. The expression of MiR-133, miR-21, and miR-143 is significantly decreased in PVAT around segments with occlusive coronary plaques (Marketou et al., 2023). The expression of pro-inflammatory miR-103-3p was higher in coronary PVAT of CAD patients, while PVAT-derived miR-382-5p suppressed foam cell formation (Vacca et al., 2016; Liu et al., 2022).

Microvessels present in the adventitia of blood vessels (vasa vasorum; VV) develop in the adventitia of atherosclerotic lesions due to hypoxic stimulation, destroy the tunica media, invade into the plaque, and play a role in inflammatory cell infiltration (Moreno et al., 2004; Phillippi, 2022). These newly proliferated blood vessels within the plaque lack pericytes and are prone to rupture, resulting in intraplaque hemorrhage (Sluimer et al., 2009; Phillippi, 2022). In this way, inflammation from the adventitia side spreads to the luminal side, promoting plaque progression and instability. Adipocytokines secreted by perivascular adipose tissue (PVAT) are thought to infiltrate directly into the vascular wall and also enter the plaque using the VV as a conduit (Sacks and Fain, 2007; Tanaka et al., 2020; Wang et al., 2022) (Right side of Figure 1).

## 4 Relationship between inflammation in EAT and CVD

It is reported that EAT in patients undergoing coronary artery bypass graft (CABG) highly expressed several inflammatory cytokines and chemokines, such as interleukin (IL)-6 and tumor necrosis factor (TNF)-α compared with their subcutaneous fat (Mazurek et al., 2003) or compared with EAT in non-CAD patients (Hirata et al., 2011b). Our observational studies revealed that EAT volume, macrophage and inflammatory cytokine content in EAT, and the decrease in adiponectin expression in EAT were risk factors for coronary artery disease requiring CABG (Shimabukuro et al., 2013).

Some clinical studies examining the relationship between EAT levels and blood biomarkers have found that there was an inverse correlation with the blood concentration of apeline, which has a vasodilatory effect (Babapour et al., 2024), and a positive correlation with the blood concentration of branched-chain amino acids (Zhao et al., 2023). Recent clinical research has shown that EAT thickness and/or volume, including those calculated using AI-based deep learning, are predictive factors for increased coronary artery lipid plaque volume (Amangurbanova et al., 2024), the onset of CVD such as coronary artery disease (Mahabadi et al., 2013; Christensen et al., 2019b; Commandeur et al., 2020; Eisenberg et al., 2020; Filtz et al., 2024; Gaborit et al., 2024; Rämö et al., 2024), and MACE (Guglielmo et al., 2024).

In recent basic and translational research on EAT, pan-genomic microarray analysis has shown that EAT have a profile similar to that of beige adipocytes (Doukbi et al., 2024). In mouse models, it has been confirmed that EAT accumulation affects the inflammatory phenotype of cardiac macrophages and induces microvascular occlusion (MVO) (Zhao et al., 2024). Recently, single-cell transcriptome analysis of human normal and pathological EAT tissues was performed, which might provide further detailed mechanisms serve as future therapeutic targets (Liu X. et al., 2024).

Furthermore, our previous studies evaluated the relationship of local inflammation in EAT, intraplaque microluminal structure (determined by optical coherence tomography; OCT) and coronary plaque characteristics (determined by integrated backscatter intravascular ultrasound; IB-IVUS) in fresh cadavers. The results showed that coronary arteries with intraplaque microluminal structure had relatively lipid-rich plaques and increased expression of the inflammatory molecules in EAT compared to coronary arteries without microluminal structure (Ito et al., 2020; Kawabata et al., 2023), suggesting the presence of luminal structures within plaques contribute to plaque instability.

## 5 Quantification of inflammation in coronary artery PVAT

In 2017, anti-inflammatory therapy targeting the IL-1β immune pathway with canakinumab for secondary prevention of myocardial infarction significantly reduced the recurrence rate of cardiovascular events, independent of lower lipid levels (CANTOS study), suggesting the relationship between chronic inflammation and CVD (Ridker et al., 2017). Also in 2017, it was reported that the state of inflammation in coronary artery PVAT can be evaluated using the fatty line attenuation coefficient (FAI), which can be obtained from coronary artery CT images (Antonopoulos et al., 2017). Computed tomography is useful for characterization as morphological changes associated with inflammation can be detected by the gradient of CT signal attenuation in PVAT [-190 to −30 Hounsfield units (HU)] (Antonopoulos et al., 2017; Theofilis et al., 2022). Indeed, lipolysis in PVAT adipocytes and adipocyte dedifferentiation induced by proinflammatory cytokines lead to an increase in the water/lipid ratio in PVAT close to the inflamed vessel wall. Consequently, these morphological changes increase the PVAT mean CT attenuation towards −30 Hounsfield units (HU) (Antonopoulos et al., 2017). The FAI of coronary PVAT in patients with CAD was consciously higher than in patients without CAD. Currently, FAI is considered to be an imaging index that reflects changes in the size of perivascular adipocytes during inflammation, and its clinical application is expected (Antonopoulos et al., 2017).

## 6 Association between perivascular FAI of coronary artery and CVD

The group above mentioned conducted two independent prospective cohort studies of symptomatic patients who underwent coronary artery CT examination to investigate the association between FAI at the root of the right coronary artery, which was most strongly associated with event onset, and all-cause mortality and cardiac death (CRISP-CT study) (Oikonomou et al., 2018). In both cohorts, FAI at the root of the right coronary artery was associated with all-cause and cardiac death, independent of conventional risk factors such as age, sex, and cardiovascular risk factors; The cutoff value was −70.1 Hounsfield units (HU), which was confirmed in the validation cohort. This group also proposed a new method using artificial intelligence (AI) to predict cardiovascular risk by analyzing the radiomic profile obtained from radiographic images of coronary artery PVAT (Oikonomou et al., 2019).

Accumulating recent clinical research have suggested a correlation between coronary artery FAI and control level of diabetes (Liu Y. et al., 2024), control level of dyslipidemia (Feng et al., 2024), coronary flow reserve (CFR) (Chen et al., 2024), plaque instability (Sagris et al., 2022; Antonopoulos and Simantiris, 2023; Kuneman et al., 2023; Wang et al., 2024), future percutaneous coronary intervention (He et al., 2024), incidence of coronary restenosis (Qin et al., 2022), and coronary bypass graft occlusion (Han et al., 2024; Huang et al., 2024). It should be noted that there may be differences between men and women regarding the characteristics of FAI (Kinoshita et al., 2024). Although many retrospective and prospective observational studies have suggested a correlation between coronary FAI and future incidence of MACE (Ichikawa et al., 2022; Kato et al., 2022; Chan et al., 2024; Choi et al., 2024; Zhan et al., 2024), there are also negative reports (van Rosendael et al., 2024; Yang et al., 2024). Alternatively, there is also literature showing that lesion-specific pericoronary FAI is a predictive factor for MACE (Liu M. et al., 2024). Currently, a large-scale prospective cohort study is underway in a multiethnic and multinational country in Asia, and will evaluate the association between AI-based coronary artery FAI and clinical endpoints such as cardiovascular events, hospitalization, and mortality (Baskaran et al., 2024). The 2023 European Society of Cardiology Recommendation document summarizes imaging methods for EAT and coronary PVAT, including FAI (Antoniades et al., 2023).

## 7 Treatments targeting EAT and coronary PVAT

If accumulation of EAT, coronary PVAT, is associated with cardiovascular events, treatment targeting PVAT resuction may potentially suppress the onset of CVD. We summarize treatments in which EAT, including coronary PVAT, is described as the potential therapeutic target.

### 7.1 Aerobic exercise

In a larger, single-center, double-blind, randomized exercise intervention study, 52 abdominally obese but otherwise healthy participants randomly assigned to moderate/high-intensity aerobic exercise (3 sessions of 45 min per week reaching 70%–85% of Vo2max) or no exercise with or without monthly infusions of the IL-6 receptor antagonist tocilizumab in the clinical standard dose of 8 mg/kg or saline (placebo). When comparing the EAT volume evaluated by MRI, a clear decrease in EAT volume was observed in the aerobic exercise group, but this effect was not observed in the tocilizumab administration group, suggesting the decrease in EAT volume due to aerobic exercise was mediated by IL-6 (Christensen et al., 2019a). A meta-analysis of five randomized controlled trials that observed whether exercise reduced EAT found that exercise significantly reduced EAT and waist circumference (Colonetti et al., 2021). The exercises used in these studies included in the meta-analysis were aerobic exercise and/or resistance circuit training, and the exercise frequency was 2–3 times a week, so exercises of such kind, intensity, and frequency might be incorporated into clinical recommendations to manage CVD risks associated with EAT. However, this report has limitations, such as insufficient blinding in the original study, and further research needs to be conducted with a more appropriate design and method to clarify the relationship between exercise and EAT. Specifically, further comparative verifications are required, including randomized controlled trials with a larger number of subjects, comparisons of exercise intensity and frequency, evaluation of the volume and feature of EAT using various imaging tests, and long-term cardiovascular prognosis. As an underlying mechanism, single-nucleus transcriptomics of epicardial adipose tissue from female pigs revealed the effects of exercise training on resident innate and adaptive immune cells (Ahmad et al., 2024).

### 7.2 Statin

In a sub-analysis of the BELLES study, which investigated whether moderate to high-dose statin administration improves coronary artery calcification in postmenopausal women, EAT volume was measured using CT. In the high-dose (atorvastatin 80 mg) group, a statistically significant decrease in EAT volume was observed after 1 year, and this was independent of the lipid-lowering effect (Alexopoulos et al., 2013). It was also reported that the FAI of PVAT in coronary arteries with non-calcified plaques was significantly reduced by 1 year of statin administration (Dai et al., 2020). Based on these results, the effects of statins on CVD may also include reducing EAT volume and suppressing inflammation.

### 7.3 SGLT2 inhibitor

Our research group compared the thickness of the EAT in type 2 diabetic patients by using echocardiography before and after treatment with canagliflozin, a SGLT2 (sodium-glucose co-transporter 2) inhibitor. The results showed that EAT thickness was significantly reduced, independent of the reduction in HbA1c levels (Yagi et al., 2017). Similar effects have been reported with ipragliflozin (Fukuda et al., 2017), luseoflozin (Bouchi et al., 2017), and dapagliflozin (Sato et al., 2018). Although the scale of individual observational studies and randomized controlled trials was small and some results were controversial, recent meta-analyses have shown that SGLT2 inhibitors significantly reduce EAT volumes in patients with type 2 diabetes and obesity (Cinti et al., 2023; Myasoedova et al., 2023; Bao et al., 2024). As for the more basic pathophysiology, recent basic research has revealed that empagliflozin suppresses the differentiation and maturation of human epicardial adipocytes, and this mechanism is at least partially responsible for the reduction in EAT levels induced by SGLT2 inhibitors (Takano et al., 2023).

### 7.4 GLP-1 receptor agonist

Recently, the efficacy and safety of glucagon-like peptide (GLP)-1 receptor agonists for the treatment of type 2 diabetes have been reported (Karagiannis et al., 2024). Some reports have shown that liraglutide, a GLP-1 receptor agonist, reduced EAT volume in type 2 diabetes (Dutour et al., 2016; Iacobellis et al., 2017), although others have shown no significant changes (van Eyk et al., 2019; Bizino et al., 2020). In recent years, many meta-analyses integrating observational studies and RCTs have been conducted, and it has been shown that GLP-1 receptor agonists significantly reduce the amount of EAT in obese patients and type 2 diabetic patients (Berg et al., 2022; Akoumianakis et al., 2023; Myasoedova et al., 2023; Bao et al., 2024). Furthermore, a recent retrospective observational study revealed an independent association between coronary FAI and semaglutide treatment, which is a widely used GLP-1 analogue (Li et al., 2024). The substudy of the randomized controlled trial (SUMMIT trial) also demonstrated that tirzepatide therapy, a commonly used GLP-1 receptor agonist, in obesity-related HFpEF led to reduced LV mass and EAT as compared to placebo (Kramer et al., 2024).

### 7.5 Anti-inflammatory drug]

Recently, it has been reported that anti-inflammatory drugs such as canakinumab (CANTOS trial) (Ridker et al., 2017) or small doses of colchicine (Nidorf et al., 2020) reduced coronary events in patients with chronic CAD. The former, the CANTOS trial, is a randomized controlled trial of canakinumab, an anti-IL-1β therapy, versus placebo for the secondary prevention of patients with myocardial infarction. Canakinumab significantly reduced the recurrence rate of cardiovascular events, independent of lower lipid levels, although canakinumab was associated with a higher incidence of fatal infection than was placebo (Ridker et al., 2017). The latter study of low-dose colchicine (0.5 mg once daily) was a randomized controlled trial versus placebo for major cardiovascular events in patients with chronic coronary artery disease. In this trial, the risk of cardiovascular events was significantly lower in patients who received colchicine than in patients who received placebo, whereas the incidence of death from non-cardiovascular causes was significantly lower than in patients who received placebo (Nidorf et al., 2020). Inflammation in EAT may be a target for such anti-inflammatory drugs. It is expected that the relationship between anti-inflammatory drugs and FAI will be elucidated through randomized clinical trials in the future. However, they also need to be carefully evaluated, including concerns about potential adverse effects such as infections due to their anti-inflammatory properties.

## 8 Discussion

In this review, we have summarized recent findings regarding the role of EAT and PVAT in the pathogenesis of atherosclerosis. Adipose tissue serves not only as an energy storage or mechanical cushion, but also as an endocrine organ. Recent evidence has revealed that perivascular adipose tissue is involved in vascular homeostasis and adjacent arterial pathophysiology by producing various adipokines (Soltis and Cassis, 1991; Löhn et al., 2002). EAT is located between the surface of the heart and the visceral layer of the pericardium and surrounds the coronary arteries. Many clinical studies suggest that an increase in EAT volume is associated with CAD (Dagvasumberel et al., 2012; Shimabukuro et al., 2013; Hirata et al., 2015; Yamada and Sata, 2015). We also reported that inflammation is enhanced in the EAT of patients with CAD (Hirata et al., 2011a; Hirata et al., 2011b), suggesting that EAT plays a crucial role in the pathogenesis of coronary atherosclerosis (Tanaka and Sata, 2018). It has been reported that exercise and some antidiabetic drugs can reduce EAT volume. Although this article mainly discussed coronary artery disease, EAT has also been reported to be associated with atrial fibrillation and heart failure (Tanaka and Sata, 2018; Elsanhoury et al., 2021). EAT and coronary PVAT may become new therapeutic targets or part of existing therapeutic targets. In particular, the CVD suppressing effects of anti-inflammatory drugs are attracting attention, and elucidation of the relationship between EAT and inflammation is expected in the future.

## 9 Conclusion

Accumulating evidence suggests that EAT, coronary PVAT, may represent new prognostic factors, new therapeutic targets, or part of existing therapeutic targets. In particular, the CVD suppressing effects of anti-inflammatory drugs are attracting attention, and elucidation of the relationship between EAT and inflammation is expected in the future.
